# COX7B Is a New Prognostic Biomarker and Correlates with Tumor Immunity in Esophageal Carcinoma

**DOI:** 10.1155/2023/6831695

**Published:** 2023-03-29

**Authors:** Yinsen Song, Na Gao, Zhenzhen Yang, Sisen Zhang, Tanli Fan, Baojun Zhang

**Affiliations:** ^1^School of Basic Medical Sciences, Xi'an Jiaotong University, Translational Medicine Research Center, Zhengzhou People's Hospital, Zhengzhou, China; ^2^Translational Medicine Research Center, Zhengzhou People's Hospital, Zhengzhou, China; ^3^School of Basic Medical Sciences, Zhengzhou University, Zhengzhou, China; ^4^Department of Pathogenic Microbiology and Immunology, School of Basic Medical Sciences, Xi'an Jiaotong University, Xi'an, China

## Abstract

Esophageal carcinoma (ESCA) refers to the most common type of malignant tumor, which reveals that it occurs often all over the world. ESCA is also correlated with an advanced stage and low survival rates. Thus, the development of new prognostic biomarkers is an absolute necessity. In this study, the aim was to investigate the potential of COX7B as a brand-new predictive biomarker for ESCA patients. COX7B expression in pancancer was examined using TIMER2. The statistical significance of the predictive value of COX7B expression was explored. The relationship between COX7B expression and tumor-infiltrating immune cells in ESCA was analyzed by using ssGSEA. In this study, the result indicated that several types of cancers had an abnormally high amount of COX7B. COX7B expression in samples from patients with ESCA was considerably higher than in nontumor tissues. A more advanced clinical stage may be anticipated from higher COX7B expression. According to the findings of Kaplan-Meier survival curves, patients with low COX7B levels had a more favorable prognosis than those with high COX7B levels. The result of multivariate analysis suggested that COX7B expression was a standalone prognostic factor for the overall survival of ESCA patients. A prognostic nomogram including gender, clinical stage, and COX7B expression was constructed, and TCGA-based calibration plots indicated its excellent predictive performance. An analysis of immune infiltration revealed that COX7B expression has a negative correlation with TFH, Tcm, NK cells, and mast cells. COX7B may serve as an immunotherapy target and as a biomarker for ESCA diagnosis and prognosis.

## 1. Introduction

Esophageal carcinoma (ESCA) is one of the most frequent malignancies in the world and is the cause of a significant number of deaths annually [1]. Men had a significantly greater incidence of the condition than women did. It is also one of the prevalent malignant tumors of the digestive system in China, with squamous carcinoma and adenocarcinoma being the primary histological forms [2, 3]. Consumption of tobacco and alcohol is the primary environmental risk factor correlated with ESCA. Although there has been significant progress in recent years in the development of early diagnostic and treatment techniques for ESCA, the five-year survival rate of just 15-20% is unacceptable [4–6]. For patients diagnosed with ESCA, the development of a reliable prognostic predictor takes on a critical significance in providing exact customized therapy [7, 8]. The existing indication is the node, tumor, and metastasis (TNM) staging system that is employed for ESCA staging and prognostic prediction with the greatest frequency [9]. Besides the TNM staging system, unique and accurate prognostic indicators should be identified to create effective treatment options for ESCA.

Mammalian Cox, often termed complex IV, refers to a multiheteromeric enzyme with 13 subunits that catalyzes the reduction of molecular oxygen to water and the oxidation of cytochrome c at the final step of OXPHOS in the mitochondrial electron transport chain [10, 11]. This occurs at the end of the oxidative phosphorylation step of the electron transport chain (ETC). Within the complex, COX7B refers to a small transmembrane protein of 80 amino acids, and it is encoded by the nucleus [12]. It is linked to the four catalytic redox centers of the enzyme that were found in the mitochondrial subunits (Cox1, Cox2, and Cox3), which were encoded by the above genes. The result indicated that the structural protein cyclooxygenase 7b (COX7B), a part of complex IV of the mitochondrial electron transport chain, was a member of a protein family that may be bigger than the one that was previously believed to account for the brain tropism in mice caused by breast cancer [13, 14]. With the help of this proof-of-concept research, it is now possible to look for metabolic sensors that are responsible for cancer organotropism and could be therapeutically addressed. This is important for therapies that prevent metastasis. Currently, there has been rare information about the role of COX7B in malignancies.

There is mounting evidence to suggest that the process of tumorigenesis is intimately connected to immunological surveillance and defense mechanisms that are activated throughout the progression of the disease [15, 16]. The above functions play an important part in determining how well a patient will respond to treatment. Immunotherapy, embodied by immune checkpoint inhibitors (ICIs), has evolved into the norm of treatment for several malignancies; yet, immunotherapy is only beneficial for a limited number of patients [17, 18]. Thus, it is essential for the treatment of cancer to conduct research into the discovery of new biomarkers that may accurately predict a patient's response to immunotherapy and to create novel therapeutic approaches that combine immunotherapy with other forms of treatment. The tumor microenvironment (TME) significantly affects the prognosis of the tumor, the likelihood of survival, and the response to treatment [19, 20]. Accordingly, acquiring a better knowledge of the pathogenic impact and dynamics of various ESCA immune cells is of great significance to the development of an effective TIME-related prognostic biomarker.

Throughout the course of this study, TCGA database was adopted to investigate the expression, prognosis, and immune infiltration of COX7B in ESCA.

## 2. Materials and Methods

### 2.1. Data Collection

The Cancer Genome Atlas (TCGA), a database for cancer genomics, can be accessed at https://cancergenome.nih.gov/. This database contains genetic data on matched normal samples and more than 2,000 primary tumors. TCGA database was used to retrieve case data for our study, including mRNA expression profiles and clinical features. This database originated from the UCSC Xena platform (https://xena.ucsc.edu/). The data from matched samples from 163 ESCA and 11 normal samples were collected for additional analysis.

### 2.2. Gene Expression Analysis of COX7B in Cancers

“COX7B” was used as the variable of interest in an investigation of the “Gene DE” component of the Tumor Immune Estimation Resource 2.0 (TIMER2) (http://timer.cistrome.org) web server's tumor immune estimation resource. In TCGA datasets, the researchers explored the ways in which the COX7B gene's expression varies between malignancies and healthy tissues.

### 2.3. Survival Evaluation

The Youden index [(sensitivity + specificity) 1] was adopted to determine the ideal COX7B cut-off value. The ESCA samples were assigned to two groups in accordance with COX7B expression levels (high and low). The survival rates of the two groups were compared using the log-rank test, and the differences in survival rates were examined using the Kaplan-Meier (K-M) method. *p* < 0.05 indicated a difference with statistical significance.

### 2.4. Analysis Using GO, KEGG, GSEA, and GSVA

KEGG pathway analysis and Gene Ontology enrichment analysis were conducted for all the differentially expressed genes (DEGs) for biological processes (BP), cellular components (CC), and molecular functions (MF). We could look at the cellular and molecular functions that COX7B performs in ESCA tissues using GO analysis. GSEA and GSVA were also used to assess the potential molecular pathways of COX7B in the same tissues. The same organs underwent the above examinations. All the analyses made use of the ClusterProfiler R tool [21].

### 2.5. Immune Infiltration Analysis

We examined the infiltration of 24 immune cell types (ICTs) in tumor tissues using the ssGSEA method, a component of the Gene Set Variation Analysis (GSVA) package of the R software. According to this study, tumor growth was substantially suppressed by ICTs. The GSA assessed the absolute expression of genes in each tumor sample, which was used to calculate an enrichment score based on the marker genes of the 24 ICTs revealed in the study. The Wilcoxon rank-sum and Spearman correlation tests were used to examine the relationship between immune cells and COX7B.

## 3. Statistical Analysis

All statistical analyses were performed using R. To determine the nature of the connection that exists between clinicopathologic features and COX7B expression, a logistic regression analysis was conducted. Using the Kaplan-Meier method and the Cox regression analysis, researchers explored the relationship between clinicopathologic features and overall survival in ESCA patients. To carry out both univariate and multivariable studies of survival, the Cox regression model was utilized. A multivariate Cox analysis was used to compare the impact of COX7B expression on survival to the impact of the other variables. A value of *p* < 0.05 was regarded as significantly different.

## 4. Results

### 4.1. Pancancer Analysis of COX7B and Its Association with Clinical Factors in ESCA

**Figure 1:**
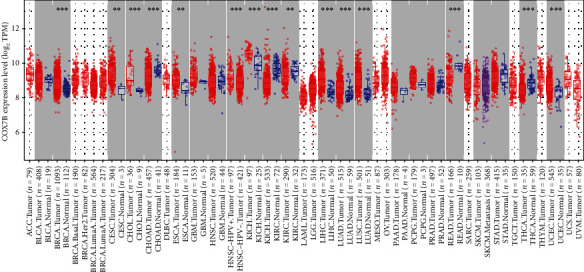
Pancancer analysis of COX7B expression.

To begin, we analyzed the COX7B expression profiles in several cancer types using data from TCGA's RNA sequencing project (including those cancers without normal tissues for comparison). According to the prediction of TIMER data, we observed that COX7B expression was higher in BRCA, CESC, CHOL, ESCA, HNSC, KICH, LIHC, LUAD, LUSC, and UCEC. Conversely, COX7B was expressed low in KIRC, KIRP, READ, and THCA (Figure 1). The full names of all tumor types are shown in Table S1.

**Figure 2:**
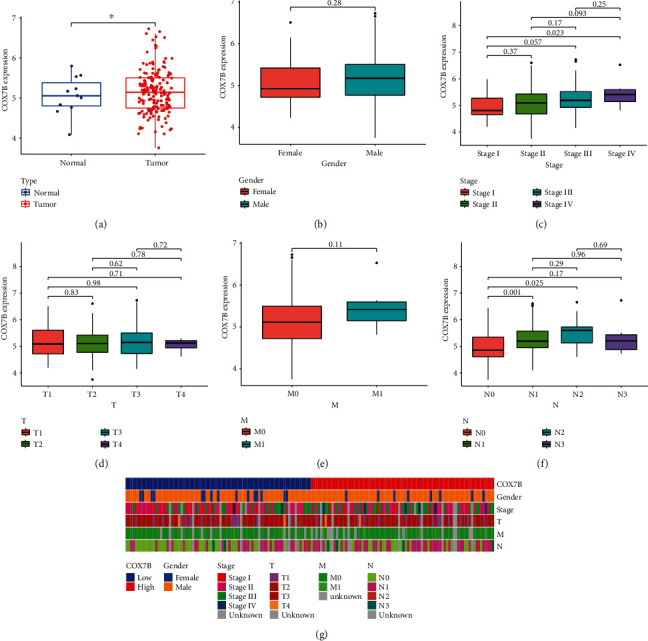
Correlations between COX7B expression in ESCA and clinical variables. (a) The distinct upregulation of COX7B was observed in ESCA specimens compared with nontumor specimens based on TCGA datasets. (b–g) Clinical features of ESCA and COX7B mRNA expression.

The histogram revealed COX7B as an overexpressed gene in ESCA samples against control samples (Figure 2(a)). Then, we analyzed its association with clinical factors in ESCA. Expression of COX7B was not correlated with either sex, as we found (Figure 2(b)). Importantly, we observed that COX7B expressed markedly higher at stage IV than stage I in ESCA (Figure 2(c)). Furthermore, there was no correlation between the T stage and M stage and COX7B expression (Figures 2(d) and 2(e)). Importantly, higher levels of COX7B were observed in the N1-N2 stage than the N0 stage (Figure 2(f)). The heatmap showed the distribution of ESCA patients with different clinical factors in the group with low or high COX7B expression (Figure 2(g)).

### 4.2. Relationship between COX7B Expression and the Clinical Outcome of ESC Patients

**Figure 3:**
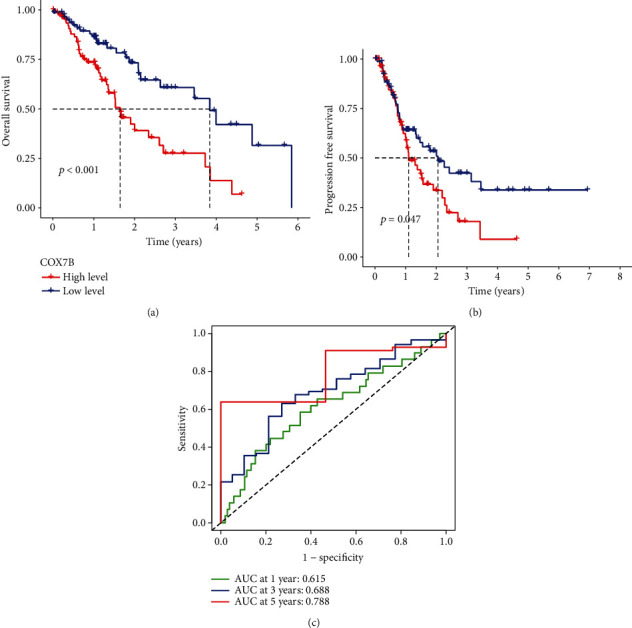
The survival analysis of COX7B in ESCA patients. (a) The OS and (b) survival distributions for PFS were plotted for patients with high and low COX7B expression in ESCA. (c) Estimating the likelihood of survival at 1, 3, and 5 years in ESCA patients using a ROC curve that changes over time.

**Figure 4:**
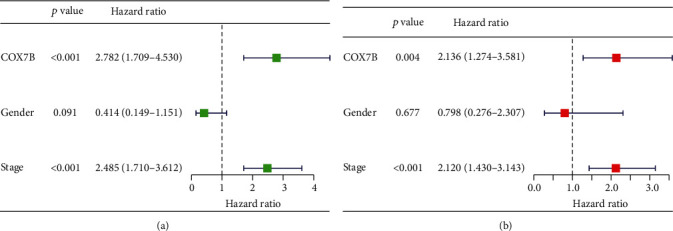
Univariate (a) and multivariate (b) analyses of prognostic factors in ESCA patients.

**Figure 5:**
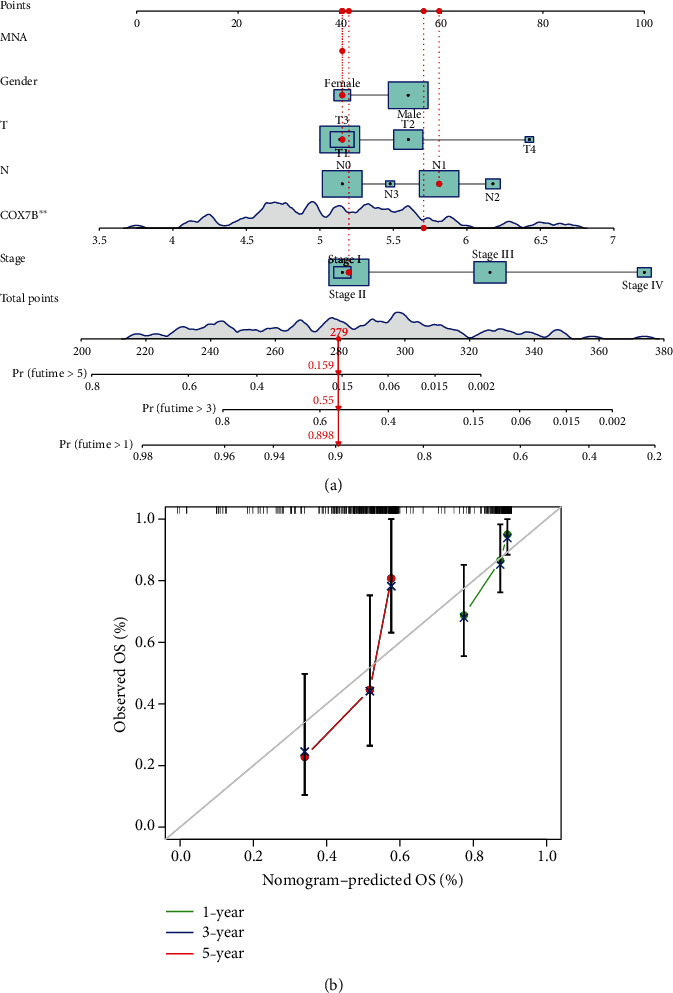
(a) Survival nomogram for estimating the likelihood of survival for ESCA patients over the course of 1, 3, and 5 years. (b) An ideal nomogram is represented by the diagonal dashed line in the calibration curve for the overall survival nomogram.

We analyzed the associations between COX7B mRNA levels and OS and PFS in patients with ESCA to investigate the potential prognostic significance of COX7B in ESCA. Patients with high COX7B mRNA expression had significantly poorer OS (*p* < 0.001, Figure 3(a)) and PFS (*p* = 0.047, Figure 3(b)) compared with the low expression group, as shown by Kaplan-Meier analysis. Based on TCGA data, COX7B expression has a high predictive capacity for the survival of ESCA patients, as indicated by an area under the ROC curve (AUC) of 0.788 (Figure 3(c)). Univariate and multivariate analyses were conducted to verify whether COX7B was an independent prognostic factor for ESCA. Clinical stage and COX7B expression were separate prognostic variables for ESCA patients (Figures 4(a) and 4(b)). A nomogram was developed using COX7B and clinical risk indicators to provide a quantitative way to predict the prognosis of ESCA patients. Clinicians now have a quantifiable tool in COX7B expression level to forecast their patients' odds of surviving 1, 3, and 5 years after initial diagnosis with ESCA (Figures 5(a) and 5(b)).

### 4.3. Enrichment Analysis

**Figure 6:**
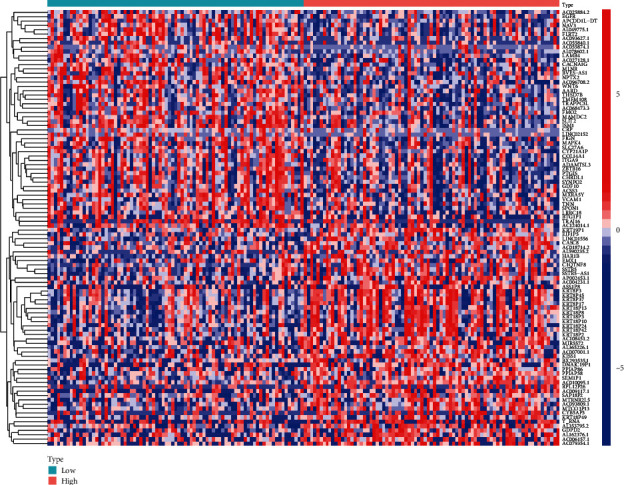
Heatmap of the DEGs between ESCA specimens with high COX7B expression and ESCA specimens with low COX7B expression.

**Figure 7:**
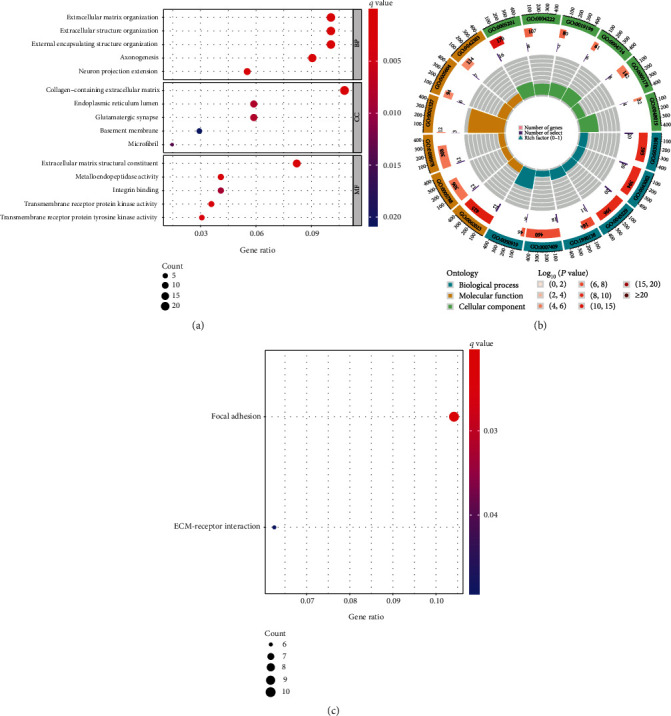
Functional enrichment analysis of DEGs in ESCA. (a, b) The GO analysis-identified biological processes with an abundance of DEGs. (c) KEGG analysis reveals enhanced pathways for DEGs.

**Figure 8:**
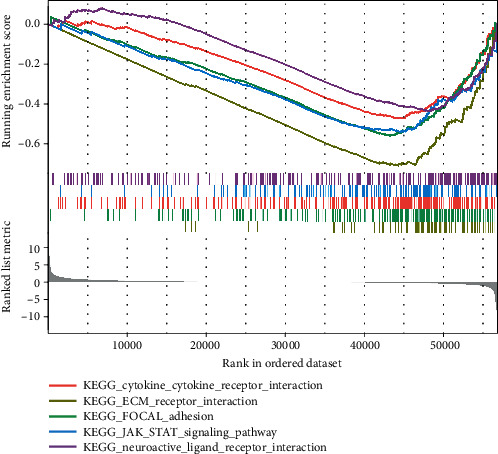
GSEA enrichment analysis of COX7B expression in ESCA patients from TCGA datasets.

To explore the potential function of COX7B in ESCA progression, we screened the differentially expressed genes (DEGs) among ESCA samples ranging from high to low COX7B expression. Finally, we screened 463 DEGs (Table S2 and Figure 6). The results of GO analysis revealed that the 463 DEGs were mainly correlated with extracellular structure organization, extracellular matrix organization, axonogenesis, external encapsulating structure organization, collagen-containing extracellular matrix, neuron projection extension, glutamatergic synapse, endoplasmic reticulum lumen, microfibril, and metalloendopeptidase activity, extracellular matrix structural constituent, and integrin binding (Figures 7(a) and 7(b)). Moreover, we performed KEGG analysis and found that the 463 DEGs were mainly enriched in focal adhesion and ECM-receptor interaction (Figure 7(c)). In addition, based on the results of the Gene Set Enrichment Analysis (GSEA), GPX1 was found to have a role in the following processes: cytokine-receptor interaction, extracellular matrix receptor interaction, focal adhesion, JAK/STAT signaling pathway, and neuroactive ligand-receptor interaction (Figure 8).

### 4.4. COX7B Expression in ESCA and the Presence of Immune Cell Infiltration

**Figure 9:**
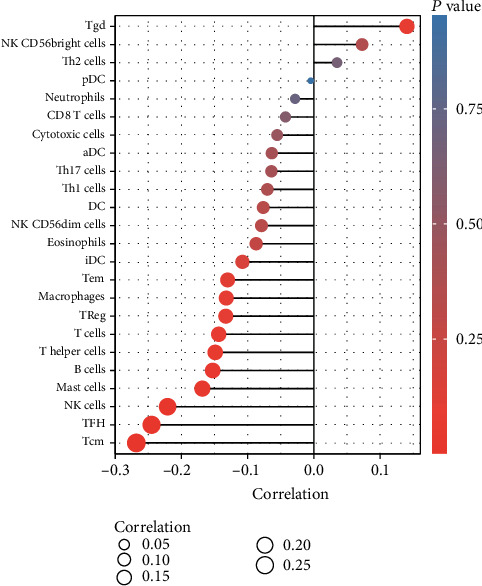
Associations of the COX7B expression level with tumor immune infiltration in ESCA. COX7B expression was inversely related to TFH, Tcm, NK cells, and mast cells.

We performed the Spearman correlation analysis to find a link between COX7B expression and immune cell infiltration in the ESCA microenvironment. It was revealed that COX7B expression was inversely related to TFH, Tcm, NK cells, and mast cells (Figure 9). The above results suggested that COX7B may be critical in controlling immune cell infiltration in the tumor microenvironment.

## 5. Discussion

In 2018, there were around 572,000 patients who were given a diagnosis of ESCA for the first time [22]. The recurrence of ESCA and the poor prognosis correlated with it continue to make it difficult to treat the condition [23, 24]. Due to the advanced stage of the disease when it is diagnosed (usually stage III or stage IV), the overall 5-year survival rate of ESCA can be as low as 20% due to the disease's high invasiveness [25, 26]. Over the course of the last couple of decades, genetic and epigenomic variables that contribute to the development of precancerous squamous lesions in the esophagus into ESCA have been the subject of an extensive amount of research and investigation [27, 28]. It has been revealed that besides cancer genetics, aberrant epigenetic regulation, which can include aberrant DNA methylation, aberrant histone modifications, and alterations of numerous noncoding RNAs, plays a crucial role in what causes and what keeps ESCA going.

Researchers have recently suggested that mutations in the COX7B gene are linked to the development of malignancies. Cox7b is a structural subunit of the mitochondrial electron transport chain (complex IV). Cox7b refers to the part of a likely wider family of proteins important for breast cancer brain tropism in mice [29]. They employed human triple-negative MDA-MB-231 breast cancer cells and two separate brain-seeking variants as models. Mice were employed in this study. This preliminary research confirmed the feasibility of a search for metabolic sensors that drive cancer organotropism and could be targeted therapeutically, which takes on a critical significance to therapies aiming at preventing metastasis. Expression of it and reports of its function were extremely infrequent in other tumor types. In this study, we performed an investigation of pancancer and revealed that COX7B displayed a dysregulated level in various types of tumors. This finding suggests that COX7B may play a role in the progression of malignancies. Because the level of COX7B expression varied in accordance with the variety of cancers, we speculated that it may act either as a tumor promotor or a tumor suppressor. We confirmed that COX7B expression is significantly greater in ESCA samples than in control samples. Patients with high COX7B expression were correlated with lower overall survival and progression-free survival compared to those with low COX7B expression, according to the results of the survival research. It is important to note that multivariate analysis indicated that COX7B expression served as an independent prognostic factor for ESCA patients. Based on our findings, COX7B may serve as an innovative predictive biomarker for patients with ESCA.

Immunotherapy with immune checkpoint inhibitors has achieved promising results in treating various cancers [30]. Significant advancements have also been made in treating advanced ESCA thanks to the use of monoclonal antibodies targeting PD-1 or PD-L1 in combination with angiogenesis inhibitors or TKIs [31]. On the other hand, there are just a few patients that have a satisfactory response to treatment. Accordingly, clarifying the immunological heterogeneity of ESCA will help doctors determine which patients are most likely to benefit from immunotherapy, and it will make it easier to screen synergistic therapeutic targets, thus increasing the efficacy of treatment. The result indicated an inverse relationship between COX7B expression and TFH, Tcm, NK cells, and mast cells. Previous research indicated that immune-inhibited cell types (e.g., reduced CD8+ T cells and M2 macrophages) were present in high numbers in ESCA [32, 33]. Undifferentiated M0 macrophages may develop into usually activated M1 macrophages, which have a phenotype that is proinflammatory and antitumorous, as revealed by the findings of a previous study [34, 35]. Besides, it has the potential to differentiate into alternatively activated macrophages (M2) that have an immune-inhibited and protumoral character. According to the results of this study, a possible prognostic indicator for ESCA is COX7B involved in immune cell infiltration. Individuals who have a low expression of COX7B and are undergoing tumor immunotherapy for ESCA may benefit more from this medication than other patients.

This study has some important caveats and restrictions. First, TCGA cohorts were mined for data used to build the diagnostic and prognostic models; however, not all the clinical parameter information may have been captured. The above cohorts were adopted to collect the data that was employed. Because of this, the outcomes may have varied from what was expected. Second, we did not give any direct in vivo proof that the COX7B upregulation had any consequences that promoted the development of cancer. As a result, an additional study should be conducted using more advanced in vivo models (e.g., a knockout mouse).

## 6. Conclusion

COX7B may be a unique prognostic biomarker and a possible therapeutic target for ESCA patients. This study underlined the clinical value of COX7B in ESCA and analyzed the effect of COX7B on immune infiltration in the tumor microenvironment.

## Data Availability

The data used to support the findings of this study are included in the article.

## Supplementary Materials

Supplementary 1 Table S1: the full names of all tumor types.

Supplementary 2 Table S2: the differentially expressed genes between ESCA specimens with high COX7B expression and ESCA specimens with low COX7B expression.
