# Supplementary material for: COX7B Is a New Prognostic Biomarker and Correlates with Tumor Immunity in Esophageal Carcinoma
Source: Mediators Inflamm. 2023 Mar 29;2023:6831695. doi: 10.1155/2023/6831695 (PMC10234374; doi:10.1155/2023/6831695)
Supplement: Supplementary 2 — Table S2: the differentially expressed genes between ESCA specimens with high COX7B expression and ESCA specimens with low COX7B expression. [file 6831695.f2.docx]

Table S2 The differentially expressed genes between ESCA specimens with high COX7B expression and ESCA specimens with low COX7B expression.

| gene | lowMean | highMean | logFC | pValue | fdr |
| --- | --- | --- | --- | --- | --- |
| RPA3P2 | 0.048552 | 0.095141 | 0.970537 | 0.001003 | 0.018766 |
| AL353795.2 | 0.06375 | 0.13284 | 1.059187 | 7.05E-05 | 0.003412 |
| AC055840.1 | 0.02539 | 0.010853 | -1.22616 | 0.001475 | 0.023489 |
| RN7SKP74 | 0.465685 | 0.261796 | -0.83091 | 0.001131 | 0.020171 |
| ASS1P8 | 0.018511 | 0.037577 | 1.021441 | 0.005045 | 0.049472 |
| RPS18P13 | 0.325155 | 0.592565 | 0.865847 | 0.000603 | 0.013814 |
| TRAJ35 | 0.425086 | 0.234638 | -0.85732 | 0.004406 | 0.045662 |
| RPS4XP20 | 0.150123 | 0.251238 | 0.742907 | 9.72E-06 | 0.000882 |
| RPL36AP26 | 0.461412 | 0.755657 | 0.711676 | 9.95E-06 | 0.0009 |
| BTNL2 | 0.044217 | 0.024215 | -0.8687 | 0.003252 | 0.03807 |
| RPL36P16 | 0.240451 | 0.432738 | 0.84775 | 0.004394 | 0.045595 |
| AC009117.1 | 0.019781 | 0.039722 | 1.005796 | 0.003354 | 0.038682 |
| RN7SL862P | 0.09188 | 0.054486 | -0.75386 | 0.002471 | 0.032275 |
| AL731557.1 | 0.02735 | 0.016051 | -0.76891 | 0.000771 | 0.016082 |
| KRT8P37 | 0.103474 | 0.20701 | 1.00043 | 0.000814 | 0.016591 |
| ATP5PFP1 | 0.09186 | 0.160368 | 0.803879 | 0.00025 | 0.007828 |
| AC068473.3 | 0.358177 | 0.106377 | -1.75149 | 0.00189 | 0.02767 |
| KRTAP5-10 | 0.104819 | 0.173663 | 0.728396 | 0.002878 | 0.03535 |
| LINC00506 | 0.036463 | 0.021199 | -0.78245 | 0.003287 | 0.038247 |
| AL023803.3 | 0.082968 | 0.141941 | 0.774664 | 0.004982 | 0.049029 |
| NRP2 | 6.2829 | 3.76762 | -0.73778 | 0.000144 | 0.005414 |
| ZNF667 | 0.190027 | 0.102452 | -0.89125 | 0.000295 | 0.008706 |
| MXRA5Y | 0.576451 | 0.225223 | -1.35584 | 0.000104 | 0.00439 |
| AC096708.2 | 0.10731 | 0.048631 | -1.14184 | 0.000165 | 0.005923 |
| IL17RD | 1.996406 | 1.125091 | -0.82736 | 6.62E-05 | 0.00327 |
| AC027128.1 | 0.166983 | 0.06987 | -1.25695 | 0.00304 | 0.036641 |
| AL355612.1 | 0.013263 | 0.007088 | -0.90402 | 0.000817 | 0.016591 |
| DNM1P51 | 0.033215 | 0.01941 | -0.77506 | 0.002164 | 0.029923 |
| SUPT20HL1 | 0.011383 | 0.006814 | -0.74036 | 0.00052 | 0.012752 |
| AP000593.3 | 0.415635 | 0.254179 | -0.70947 | 0.002934 | 0.035795 |
| CYP2S1 | 49.85276 | 81.36 | 0.706646 | 0.001659 | 0.025281 |
| EIF1P5 | 0.832499 | 1.811998 | 1.122061 | 0.000712 | 0.015282 |
| EFHD1 | 3.73568 | 1.845101 | -1.01767 | 0.004599 | 0.046707 |
| PPIAP80 | 0.041452 | 0.072205 | 0.800661 | 0.0028 | 0.034894 |
| MIR6870 | 0.861354 | 0.433083 | -0.99196 | 0.003674 | 0.040743 |
| AC008708.2 | 0.028856 | 0.057183 | 0.986699 | 0.001703 | 0.025819 |
| AC020923.1 | 0.167864 | 0.317681 | 0.920292 | 0.002473 | 0.032275 |
| MIR548A3 | 0.263688 | 0.156454 | -0.75309 | 0.001191 | 0.020865 |
| AC090772.1 | 0.506249 | 0.308449 | -0.71481 | 0.000513 | 0.012629 |
| COX7B | 26.0371 | 49.31472 | 0.92145 | 4.41E-28 | 1.33E-23 |
| TNN | 0.530759 | 0.161142 | -1.71973 | 0.003904 | 0.042396 |
| ADORA2A-AS1 | 0.195811 | 0.099253 | -0.98028 | 0.003565 | 0.039939 |
| MN1 | 5.422427 | 2.994544 | -0.8566 | 0.001316 | 0.021913 |
| MAP9 | 1.642202 | 1.009714 | -0.70169 | 0.004623 | 0.046873 |
| DDR2 | 4.753688 | 2.852073 | -0.73704 | 0.000301 | 0.008809 |
| MTCO2P12 | 13.59884 | 22.95479 | 0.755311 | 2.10E-05 | 0.001513 |
| S1PR3 | 2.946903 | 1.717573 | -0.77883 | 0.002251 | 0.030526 |
| PPIAP86 | 0.036007 | 0.07373 | 1.033951 | 0.003517 | 0.039689 |
| MIR623 | 0.533457 | 0.283798 | -0.91051 | 0.000116 | 0.004666 |
| THBS2 | 15.20508 | 8.61972 | -0.81884 | 0.002999 | 0.036335 |
| MUSK | 0.147123 | 0.085923 | -0.7759 | 0.001978 | 0.028468 |
| BTF3P2 | 0.104481 | 0.171465 | 0.714679 | 0.000144 | 0.005439 |
| CPAMD8 | 0.78071 | 0.370388 | -1.07575 | 0.002615 | 0.033358 |
| H3C2 | 0.592541 | 1.046609 | 0.820736 | 0.000957 | 0.018185 |
| AL365226.1 | 1.709569 | 4.157499 | 1.282084 | 0.000119 | 0.004768 |
| KRT18P29 | 0.073607 | 0.131747 | 0.839858 | 0.002162 | 0.029923 |
| NPIPB6 | 0.086093 | 0.146898 | 0.770849 | 0.00188 | 0.027569 |
| AC023202.1 | 0.102225 | 0.050969 | -1.00405 | 0.005012 | 0.049248 |
| PABPC5 | 0.172805 | 0.106005 | -0.70501 | 0.004456 | 0.045846 |
| RNU6-353P | 0.799985 | 1.403247 | 0.810724 | 7.80E-06 | 0.000745 |
| PABPC4L | 0.817404 | 0.415136 | -0.97747 | 0.003342 | 0.038559 |
| RNU6-136P | 0.285808 | 0.148949 | -0.94022 | 0.00466 | 0.046998 |
| ADAMTS3 | 0.420153 | 0.236814 | -0.82716 | 0.004386 | 0.045532 |
| RPS26P43 | 0.133378 | 0.229258 | 0.781454 | 0.001252 | 0.021381 |
| ZNF625 | 0.038644 | 0.023523 | -0.71616 | 0.002875 | 0.035336 |
| APCDD1L-DT | 0.320059 | 0.102889 | -1.63725 | 0.00482 | 0.048003 |
| PRIMA1 | 3.07405 | 1.617469 | -0.9264 | 0.002824 | 0.035029 |
| AC243960.10 | 0.048149 | 0.02674 | -0.84854 | 0.002057 | 0.029086 |
| AL590396.2 | 0.015643 | 0.026733 | 0.773103 | 0.002148 | 0.029807 |
| RPS26P54 | 0.091451 | 0.165246 | 0.853548 | 0.001138 | 0.020286 |
| FHOD3 | 2.003048 | 1.013081 | -0.98345 | 0.000156 | 0.005714 |
| ADI1P1 | 0.035281 | 0.019506 | -0.85498 | 0.001015 | 0.018815 |
| LAMB4 | 0.273621 | 0.097641 | -1.48662 | 0.001278 | 0.021624 |
| KRT18P38 | 0.138093 | 0.254969 | 0.884687 | 0.000309 | 0.008978 |
| RPL36AP29 | 0.212006 | 0.413553 | 0.963966 | 0.001717 | 0.025903 |
| KRT18P2 | 0.015925 | 0.032643 | 1.035518 | 0.001894 | 0.027694 |
| AL138827.1 | 0.124897 | 0.21861 | 0.807621 | 0.000877 | 0.017351 |
| KRT18P25 | 0.097522 | 0.190422 | 0.965408 | 0.000117 | 0.004691 |
| AC013549.2 | 0.060369 | 0.113236 | 0.907462 | 0.003193 | 0.037656 |
| ST3GAL5-AS1 | 0.080373 | 0.046764 | -0.78131 | 0.000869 | 0.01724 |
| CCDC158 | 0.115626 | 0.063728 | -0.85946 | 0.004363 | 0.045356 |
| HMCN1 | 2.313752 | 1.278211 | -0.85611 | 0.000258 | 0.007946 |
| LINC01550 | 0.28147 | 0.166106 | -0.76088 | 0.0026 | 0.033217 |
| ZNF471 | 0.438516 | 0.253465 | -0.79084 | 0.002327 | 0.031117 |
| LTBP1 | 19.28646 | 11.83522 | -0.7045 | 3.90E-05 | 0.002289 |
| ZNF385B | 0.351757 | 0.180096 | -0.96581 | 0.000969 | 0.018277 |
| CX3CL1 | 11.6361 | 6.678768 | -0.80095 | 0.004456 | 0.045846 |
| BICC1 | 2.287097 | 1.382322 | -0.72642 | 0.003201 | 0.037677 |
| FBLN2 | 19.2715 | 10.6569 | -0.85468 | 0.001659 | 0.025281 |
| AC095031.1 | 0.170833 | 0.292151 | 0.774128 | 0.002006 | 0.028707 |
| FBXW7-AS1 | 0.135246 | 0.081523 | -0.7303 | 0.001846 | 0.027228 |
| EEF1B2P4 | 0.038522 | 0.074806 | 0.957466 | 0.004425 | 0.045812 |
| LINC01556 | 0.23699 | 0.520933 | 1.136276 | 6.86E-05 | 0.003369 |
| CHRDL1 | 4.432348 | 1.525841 | -1.53847 | 0.002276 | 0.030744 |
| RNFT2 | 1.007599 | 1.716167 | 0.768268 | 0.000399 | 0.010681 |
| GPC6 | 3.505615 | 2.058254 | -0.76825 | 0.002778 | 0.034719 |
| KRT8P17 | 0.049117 | 0.099746 | 1.022024 | 0.001935 | 0.028103 |
| AC093627.1 | 0.106547 | 0.04327 | -1.30004 | 0.003465 | 0.039343 |
| ADAMTS1 | 9.360562 | 5.373546 | -0.80072 | 0.003682 | 0.040743 |
| LRCH2 | 0.744219 | 0.398368 | -0.90163 | 0.000238 | 0.007611 |
| PPIAP58 | 0.133259 | 0.298457 | 1.163293 | 0.004292 | 0.045038 |
| CSPG4P13 | 0.153692 | 0.073465 | -1.0649 | 0.004845 | 0.048063 |
| PLCD4 | 0.196236 | 0.107249 | -0.87162 | 0.004999 | 0.049136 |
| BX293535.1 | 0.48617 | 1.017952 | 1.066137 | 0.003781 | 0.041543 |
| AL118556.1 | 0.22305 | 0.134902 | -0.72545 | 0.002418 | 0.031879 |
| CACNA1G | 0.095935 | 0.041122 | -1.22213 | 3.98E-05 | 0.002316 |
| AC094085.1 | 0.048914 | 0.085663 | 0.808418 | 0.001587 | 0.024636 |
| SALL2 | 1.277804 | 0.757341 | -0.75465 | 0.004999 | 0.049136 |
| LZTS1 | 3.695209 | 2.142978 | -0.78604 | 0.000183 | 0.006383 |
| KCNS2 | 0.061318 | 0.034346 | -0.83618 | 0.001566 | 0.024427 |
| AC018714.2 | 0.084946 | 0.3099 | 1.86719 | 0.000503 | 0.012465 |
| ROPN1 | 0.022247 | 0.01199 | -0.89176 | 0.00144 | 0.023068 |
| FABP6 | 1.787309 | 3.394479 | 0.925401 | 0.002081 | 0.029236 |
| ATP5MF | 28.50158 | 48.50423 | 0.767069 | 1.59E-06 | 0.00023 |
| AC004231.1 | 0.159083 | 1.353283 | 3.088614 | 0.004837 | 0.048063 |
| AC026412.4 | 0.013181 | 0.023435 | 0.830126 | 0.003987 | 0.04286 |
| TRIM9 | 0.422688 | 0.20646 | -1.03373 | 0.002238 | 0.030437 |
| ATP8B2 | 5.155578 | 2.749037 | -0.90721 | 2.10E-06 | 0.000279 |
| MIR6075 | 0.188788 | 0.089598 | -1.07523 | 0.00116 | 0.020544 |
| NECAB1 | 0.417573 | 0.241464 | -0.79022 | 0.001387 | 0.022591 |
| HSPE1P4 | 0.515906 | 0.880132 | 0.770611 | 2.63E-06 | 0.000324 |
| GNB4 | 6.930727 | 3.959642 | -0.80764 | 0.000117 | 0.004691 |
| TANC2 | 4.609828 | 2.794205 | -0.72228 | 3.37E-05 | 0.002059 |
| MACF1 | 11.72395 | 7.19474 | -0.70444 | 5.14E-06 | 0.000538 |
| CRP | 14.64296 | 0.030668 | -8.89926 | 0.002278 | 0.030758 |
| PRELID1P5 | 0.527472 | 0.899543 | 0.770097 | 3.25E-07 | 7.77E-05 |
| RNF128 | 10.15588 | 19.09417 | 0.910817 | 0.00164 | 0.025158 |
| WNT6 | 1.564538 | 0.365615 | -2.09734 | 0.003271 | 0.038085 |
| TMEM108 | 0.682477 | 0.236414 | -1.52947 | 0.003288 | 0.038247 |
| MAMDC2 | 2.432743 | 0.909309 | -1.41974 | 0.001395 | 0.022591 |
| AL163953.1 | 0.311601 | 0.514433 | 0.723286 | 0.004483 | 0.046061 |
| KRT18P16 | 0.137835 | 0.23223 | 0.752614 | 0.003218 | 0.037837 |
| RPL39P25 | 0.094325 | 0.156485 | 0.730319 | 0.004616 | 0.046865 |
| AC025165.1 | 0.076516 | 0.045352 | -0.7546 | 0.001616 | 0.024979 |
| GXYLT2 | 4.248416 | 2.203153 | -0.94736 | 0.003271 | 0.038085 |
| FGF11 | 0.178808 | 0.101665 | -0.81458 | 0.000695 | 0.015107 |
| AC006159.1 | 0.491088 | 0.251728 | -0.96411 | 0.000363 | 0.01013 |
| PEG3 | 0.105759 | 0.054507 | -0.95626 | 0.00113 | 0.020171 |
| AC106795.1 | 2.451975 | 4.26789 | 0.799579 | 2.82E-05 | 0.001826 |
| KRT8 | 112.8441 | 199.7997 | 0.824224 | 0.002658 | 0.033772 |
| AC105935.2 | 0.152816 | 0.283477 | 0.891433 | 0.001515 | 0.023921 |
| AC069271.1 | 0.482577 | 0.896533 | 0.893598 | 2.02E-05 | 0.00147 |
| TNS1 | 18.57997 | 10.57764 | -0.81273 | 0.00029 | 0.008581 |
| BVES-AS1 | 0.091676 | 0.042575 | -1.10653 | 0.003924 | 0.04247 |
| KRT18P17 | 0.167406 | 0.304394 | 0.862592 | 0.00073 | 0.01557 |
| AC113189.3 | 2.766812 | 1.45644 | -0.92578 | 0.000224 | 0.007329 |
| KRT18P10 | 0.37668 | 0.800515 | 1.08759 | 0.00019 | 0.006552 |
| SAP18P2 | 0.057509 | 0.146081 | 1.344923 | 0.000728 | 0.015553 |
| EPHA3 | 1.217924 | 0.658072 | -0.88811 | 0.000809 | 0.016537 |
| ADAMTSL3 | 0.784101 | 0.351105 | -1.15914 | 0.000691 | 0.015037 |
| SYNPO2 | 7.855535 | 3.409935 | -1.20397 | 0.002658 | 0.033772 |
| P2RY12 | 0.219352 | 0.110858 | -0.98453 | 0.003271 | 0.038085 |
| HAR1B | 0.036007 | 0.078315 | 1.121016 | 0.00345 | 0.039211 |
| RPL7P33 | 0.1104 | 0.19057 | 0.787584 | 9.64E-06 | 0.000878 |
| TLL1 | 0.864719 | 0.414768 | -1.05993 | 0.000957 | 0.018185 |
| ITGA9 | 2.831305 | 1.288158 | -1.13616 | 0.00246 | 0.032181 |
| TPTEP1 | 0.438213 | 0.214186 | -1.03277 | 0.003883 | 0.042202 |
| ANKRD6 | 0.883546 | 0.517004 | -0.77313 | 0.003048 | 0.036693 |
| AC064875.1 | 0.039482 | 0.023258 | -0.76347 | 0.003077 | 0.036866 |
| KRT18P65 | 0.063538 | 0.115031 | 0.856323 | 0.004454 | 0.045846 |
| PLPPR4 | 0.975655 | 0.557007 | -0.80867 | 0.000244 | 0.007723 |
| LINC01237 | 0.178228 | 0.105928 | -0.75064 | 0.004819 | 0.048003 |
| AC010631.1 | 0.119441 | 0.223431 | 0.903533 | 0.000582 | 0.013565 |
| AC009542.1 | 0.102057 | 0.059047 | -0.78945 | 0.003781 | 0.041543 |
| AC093423.2 | 0.064615 | 0.038356 | -0.75243 | 0.003612 | 0.040281 |
| RPL23AP57 | 0.674104 | 1.113998 | 0.724704 | 0.00098 | 0.018461 |
| CELF2 | 2.824514 | 1.651156 | -0.77453 | 0.002327 | 0.031117 |
| FERMT2 | 5.756985 | 3.507722 | -0.71478 | 0.000546 | 0.013098 |
| KRT18 | 89.93235 | 165.1172 | 0.876579 | 0.000849 | 0.016975 |
| LRFN5 | 0.17772 | 0.095735 | -0.8925 | 0.001191 | 0.020865 |
| AC106772.1 | 0.219861 | 0.374736 | 0.769281 | 0.000769 | 0.016082 |
| AC007001.1 | 0.062441 | 0.128067 | 1.036335 | 0.001196 | 0.020867 |
| RPL12P26 | 0.016677 | 0.034919 | 1.066172 | 0.003407 | 0.038952 |
| FMO2 | 7.862129 | 3.08313 | -1.35052 | 0.002934 | 0.035795 |
| AC112721.2 | 0.28204 | 0.160541 | -0.81296 | 0.000365 | 0.01013 |
| H4C3 | 1.336467 | 2.226979 | 0.736664 | 1.40E-07 | 4.29E-05 |
| FBN1 | 11.09548 | 6.349809 | -0.80519 | 0.0026 | 0.033217 |
| AC011383.1 | 0.086186 | 0.052742 | -0.70851 | 0.000871 | 0.017263 |
| SSTR5-AS1 | 0.151302 | 0.703499 | 2.217118 | 0.000227 | 0.007366 |
| EFNB3 | 1.756683 | 1.00986 | -0.7987 | 0.004317 | 0.045129 |
| MTRNR2L9 | 0.575523 | 0.974168 | 0.759297 | 0.000571 | 0.013435 |
| KRT18P8 | 0.142904 | 0.319978 | 1.162928 | 2.32E-06 | 0.000298 |
| KRT19 | 332.5512 | 635.9281 | 0.935287 | 0.000513 | 0.012629 |
| RBMX2P3 | 0.076796 | 0.12921 | 0.750608 | 0.000934 | 0.017982 |
| IRX1 | 1.416658 | 0.807426 | -0.81109 | 0.000705 | 0.015222 |
| AC016542.2 | 0.910757 | 1.703674 | 0.903511 | 0.000375 | 0.010278 |
| VLDLR | 1.927502 | 1.133725 | -0.76566 | 0.000148 | 0.005586 |
| LINC01801 | 0.158135 | 0.092415 | -0.77496 | 0.001104 | 0.019881 |
| RNU6-554P | 0.187105 | 0.361259 | 0.949187 | 0.002065 | 0.029159 |
| AC018645.2 | 0.13781 | 0.066685 | -1.04724 | 5.28E-05 | 0.00282 |
| COL8A2 | 5.623567 | 3.205904 | -0.81075 | 0.003604 | 0.040205 |
| LTBP2 | 21.20421 | 11.99325 | -0.82213 | 9.39E-07 | 0.00016 |
| MIR548P | 0.199691 | 0.102121 | -0.96749 | 0.003122 | 0.037173 |
| MMP16 | 0.498699 | 0.304564 | -0.71142 | 0.003842 | 0.041906 |
| AC012519.1 | 0.03492 | 0.06912 | 0.985055 | 0.000181 | 0.006347 |
| RTP5 | 0.045771 | 0.026569 | -0.78468 | 0.003144 | 0.037302 |
| ISM1 | 2.219446 | 1.028733 | -1.10933 | 8.88E-05 | 0.003966 |
| AL161725.1 | 0.14534 | 0.087056 | -0.73942 | 0.003967 | 0.042751 |
| MTRNR2L12 | 6.863027 | 11.28647 | 0.717678 | 0.000226 | 0.007346 |
| ARHGAP20 | 0.520702 | 0.312393 | -0.7371 | 0.001185 | 0.020792 |
| COL14A1 | 5.776652 | 2.626977 | -1.13683 | 0.001945 | 0.028192 |
| AL450163.1 | 0.077808 | 0.131228 | 0.754089 | 0.002542 | 0.032713 |
| MTRNR2L5 | 0.010594 | 0.022811 | 1.106513 | 0.000598 | 0.013775 |
| AC010095.1 | 0.226373 | 0.518885 | 1.196714 | 7.59E-09 | 4.38E-06 |
| S100P | 164.2386 | 275.9042 | 0.748374 | 0.003452 | 0.039211 |
| SSTR5 | 0.523931 | 1.219083 | 1.218346 | 3.77E-05 | 0.002246 |
| SLIT3 | 3.335025 | 1.786023 | -0.90095 | 0.000662 | 0.01467 |
| AC040169.1 | 1.153033 | 1.876094 | 0.702299 | 0.000775 | 0.016123 |
| CYB5AP3 | 0.016948 | 0.042111 | 1.313126 | 0.00261 | 0.03331 |
| PDCD5P1 | 0.355347 | 0.604423 | 0.766331 | 2.53E-05 | 0.001723 |
| AC011933.1 | 1.941436 | 3.238944 | 0.738399 | 8.26E-07 | 0.000145 |
| AP002453.1 | 0.073205 | 0.151254 | 1.046963 | 0.001374 | 0.0225 |
| EIF1 | 149.8511 | 244.8818 | 0.708556 | 0.001316 | 0.021913 |
| RN7SKP26 | 0.293459 | 0.144537 | -1.02172 | 0.002351 | 0.031271 |
| KRT18P23 | 0.067279 | 0.1281 | 0.929042 | 0.000986 | 0.018547 |
| Y_RNA | 0.081094 | 0.161457 | 0.993484 | 0.001882 | 0.027579 |
| ATP5MDP1 | 1.071043 | 1.770732 | 0.725329 | 6.06E-09 | 3.97E-06 |
| EGFR | 68.89741 | 25.90749 | -1.41108 | 0.000574 | 0.013475 |
| SEC24AP1 | 0.065811 | 0.033436 | -0.97694 | 0.003617 | 0.040321 |
| DDX39B-AS1 | 0.080063 | 0.135711 | 0.761332 | 0.004571 | 0.046563 |
| RN7SL417P | 0.562091 | 0.311841 | -0.84999 | 0.002622 | 0.033443 |
| AC079354.1 | 0.043091 | 0.131551 | 1.610148 | 0.000102 | 0.004333 |
| HOXC-AS3 | 0.220346 | 0.358169 | 0.700872 | 0.003738 | 0.041233 |
| GIMAP3P | 0.03533 | 0.016953 | -1.05936 | 0.000252 | 0.00784 |
| KCNMA1 | 0.96696 | 0.464959 | -1.05635 | 0.002406 | 0.031736 |
| SLC27A6 | 0.761606 | 0.347717 | -1.13113 | 0.001361 | 0.022373 |
| RN7SL3 | 2.528869 | 4.230014 | 0.74217 | 0.003642 | 0.040503 |
| CAMK4 | 0.549172 | 0.325717 | -0.75364 | 0.000358 | 0.010002 |
| SEM1P1 | 0.770025 | 1.65207 | 1.101299 | 4.90E-06 | 0.00052 |
| RPS15AP30 | 0.522151 | 0.868579 | 0.734189 | 0.001478 | 0.023496 |
| AC004264.2 | 0.172335 | 0.091137 | -0.9191 | 0.002212 | 0.030195 |
| LRRK2 | 1.079275 | 0.57176 | -0.91658 | 0.00079 | 0.016285 |
| PAPLN | 3.792397 | 2.100909 | -0.8521 | 0.000188 | 0.006503 |
| FLRT2 | 0.981257 | 0.395462 | -1.31109 | 0.000157 | 0.005738 |
| AL672212.1 | 0.062165 | 0.102022 | 0.714713 | 0.000123 | 0.004894 |
| AC087477.1 | 0.21068 | 0.342317 | 0.700279 | 0.001293 | 0.021724 |
| C8orf88 | 0.834465 | 0.396764 | -1.07257 | 0.000691 | 0.015037 |
| AC006116.9 | 0.048 | 0.027201 | -0.81936 | 0.004334 | 0.045257 |
| AC079316.2 | 0.16979 | 0.090754 | -0.90371 | 0.001058 | 0.019423 |
| KRT18P11 | 0.279821 | 0.543672 | 0.958231 | 0.000482 | 0.01211 |
| KRT8P32 | 0.13583 | 0.239442 | 0.817878 | 0.001185 | 0.020792 |
| SNHG25 | 8.978598 | 14.63376 | 0.704739 | 0.000325 | 0.009336 |
| XCR1 | 0.43098 | 0.208825 | -1.04533 | 0.001249 | 0.021346 |
| KF455155.1 | 0.056908 | 0.033293 | -0.77343 | 0.000937 | 0.018013 |
| RPL39P29 | 0.131209 | 0.220232 | 0.747162 | 0.004554 | 0.046427 |
| CRISPLD1 | 2.742716 | 1.503454 | -0.86732 | 5.20E-05 | 0.002808 |
| AC093635.1 | 0.224831 | 0.382923 | 0.768212 | 0.000844 | 0.016917 |
| RPS28P4 | 17.28954 | 28.72533 | 0.732424 | 2.13E-05 | 0.001532 |
| AC104232.3 | 0.062383 | 0.032798 | -0.92756 | 0.002322 | 0.031117 |
| SPARCL1 | 58.0851 | 35.55176 | -0.70825 | 0.003201 | 0.037677 |
| SORCS2 | 1.61372 | 0.948294 | -0.76698 | 0.000394 | 0.010593 |
| LINC02189 | 0.049909 | 0.02888 | -0.78922 | 0.000482 | 0.01211 |
| MAB21L1 | 0.169794 | 0.09308 | -0.86724 | 0.002196 | 0.030122 |
| KRT8P1 | 0.024341 | 0.040399 | 0.730938 | 0.004032 | 0.043128 |
| TOX | 2.850235 | 1.413089 | -1.01223 | 0.001363 | 0.022373 |
| GLI3 | 3.19927 | 1.844248 | -0.79471 | 0.002999 | 0.036335 |
| TRAJ16 | 0.392179 | 0.148344 | -1.40256 | 0.004633 | 0.046883 |
| ANKRD31 | 0.14997 | 0.074384 | -1.01161 | 0.002366 | 0.03139 |
| AC092067.1 | 0.208869 | 0.344357 | 0.721305 | 0.002127 | 0.029652 |
| PCDH18 | 4.144703 | 2.530586 | -0.7118 | 0.003565 | 0.039939 |
| PDE10A | 0.432249 | 0.260253 | -0.73195 | 0.003988 | 0.04286 |
| APOO | 4.239072 | 7.034231 | 0.730644 | 5.42E-11 | 1.09E-07 |
| BOC | 2.281151 | 1.13769 | -1.00365 | 0.000156 | 0.005714 |
| RASSF8 | 3.844265 | 2.245443 | -0.77571 | 0.002081 | 0.029236 |
| KRT18P13 | 0.071373 | 0.15476 | 1.11659 | 3.70E-06 | 0.000416 |
| CCDC144B | 0.294702 | 0.16506 | -0.83626 | 0.003452 | 0.039211 |
| LINC01952 | 0.126217 | 0.228921 | 0.858947 | 0.000945 | 0.018072 |
| AL714022.1 | 0.30403 | 0.511951 | 0.75179 | 0.001835 | 0.027122 |
| COX17P1 | 1.69539 | 2.943601 | 0.795965 | 1.43E-05 | 0.001159 |
| BVES | 1.667324 | 0.928196 | -0.84503 | 0.000365 | 0.01013 |
| HSPE1P6 | 0.444138 | 0.730626 | 0.718126 | 0.000152 | 0.005637 |
| DZIP1 | 2.312828 | 1.380001 | -0.74499 | 0.003642 | 0.040503 |
| AL449983.1 | 0.080151 | 0.048814 | -0.71543 | 0.001606 | 0.02485 |
| CYP21A1P | 0.252916 | 0.086035 | -1.55567 | 0.003411 | 0.038952 |
| MLNR | 0.149402 | 0.064905 | -1.2028 | 0.000931 | 0.017982 |
| AC243562.1 | 0.42984 | 0.238899 | -0.8474 | 0.004885 | 0.048423 |
| KRT18P54 | 0.04997 | 0.098326 | 0.976499 | 0.003009 | 0.036443 |
| BTG1P1 | 0.074967 | 0.03229 | -1.21517 | 0.00468 | 0.047108 |
| AC112721.1 | 0.216725 | 0.1255 | -0.78818 | 0.002825 | 0.035029 |
| CLEC1B | 0.061366 | 0.030652 | -1.00147 | 0.001726 | 0.025984 |
| HSPE1P7 | 0.20488 | 0.382842 | 0.901968 | 2.65E-06 | 0.000324 |
| DPYS | 0.03844 | 0.023554 | -0.70662 | 0.001204 | 0.020951 |
| AC025884.2 | 0.667275 | 0.173723 | -1.94149 | 0.003049 | 0.036693 |
| KRT8P7 | 0.129598 | 0.254268 | 0.972311 | 0.001566 | 0.024427 |
| H4C13 | 0.097946 | 0.163981 | 0.743479 | 0.00434 | 0.045257 |
| AL078602.1 | 0.025269 | 0.011135 | -1.1823 | 0.000689 | 0.015037 |
| EBP | 7.812322 | 13.61238 | 0.801096 | 5.16E-09 | 3.45E-06 |
| ZDBF2 | 1.75684 | 1.00551 | -0.80506 | 0.0026 | 0.033217 |
| KRT8P40 | 0.124432 | 0.211522 | 0.765451 | 0.002431 | 0.031992 |
| MAGIX | 0.729468 | 1.197654 | 0.715295 | 0.000504 | 0.012465 |
| PDGFC | 4.602755 | 2.285512 | -1.00998 | 9.26E-05 | 0.004075 |
| VCAN | 15.00956 | 9.23692 | -0.7004 | 0.003378 | 0.038729 |
| GALNT17 | 0.791088 | 0.391938 | -1.01321 | 0.001104 | 0.019881 |
| ACSS3 | 0.251586 | 0.111632 | -1.1723 | 0.004479 | 0.04604 |
| AL590135.1 | 0.835659 | 1.553489 | 0.894526 | 1.35E-07 | 4.18E-05 |
| MAPK4 | 0.732375 | 0.144356 | -2.34296 | 0.003178 | 0.037531 |
| LAMA2 | 2.448825 | 1.293642 | -0.92065 | 0.000384 | 0.010439 |
| GAS7 | 3.901261 | 2.229858 | -0.80699 | 0.001016 | 0.018815 |
| KCNT2 | 0.358351 | 0.180043 | -0.99303 | 0.000284 | 0.008525 |
| DYNC2H1 | 1.44633 | 0.774265 | -0.9015 | 0.00078 | 0.016166 |
| DNAJC19P1 | 0.046043 | 0.110427 | 1.262054 | 0.002514 | 0.032548 |
| AC008522.1 | 0.013985 | 0.024169 | 0.789267 | 0.001242 | 0.021262 |
| LINGO3 | 0.472463 | 0.285677 | -0.72582 | 0.004871 | 0.048298 |
| CDK14 | 4.091042 | 2.494816 | -0.71353 | 0.001756 | 0.026236 |
| MIEN1 | 34.68773 | 64.70613 | 0.899477 | 2.65E-06 | 0.000324 |
| AC018868.1 | 2.823709 | 4.778104 | 0.758847 | 3.33E-06 | 0.000381 |
| TMEM244 | 0.075299 | 0.037789 | -0.99468 | 0.003083 | 0.036866 |
| LRP4-AS1 | 0.16162 | 0.27727 | 0.77869 | 0.002629 | 0.033499 |
| TUBB4BP3 | 0.092375 | 0.174795 | 0.920095 | 0.002054 | 0.029086 |
| CDKN2B | 13.7772 | 7.870858 | -0.80769 | 0.003098 | 0.036917 |
| RPL23AP35 | 0.047874 | 0.079435 | 0.730522 | 0.001579 | 0.024603 |
| GPR88 | 0.114621 | 0.055483 | -1.04676 | 0.00341 | 0.038952 |
| AC093809.1 | 2.27233 | 4.611686 | 1.021122 | 5.27E-05 | 0.00282 |
| ABI3BP | 2.554895 | 1.423633 | -0.84369 | 0.002177 | 0.029923 |
| SPON1 | 9.713769 | 4.323906 | -1.1677 | 0.002516 | 0.032548 |
| KRT8P20 | 0.055419 | 0.099779 | 0.848368 | 0.003842 | 0.041906 |
| KRT17P4 | 0.006676 | 0.011977 | 0.843172 | 0.004506 | 0.046126 |
| PTGIS | 5.03693 | 2.295791 | -1.13355 | 0.000286 | 0.008538 |
| BTBD9-AS1 | 0.458662 | 0.807289 | 0.815655 | 0.001234 | 0.021241 |
| H2AC14 | 0.417718 | 0.752504 | 0.84917 | 0.000464 | 0.01184 |
| AL024508.1 | 1.327098 | 2.279616 | 0.780516 | 0.002251 | 0.030526 |
| SETBP1 | 2.019894 | 1.167314 | -0.79109 | 0.000114 | 0.004619 |
| AP001086.1 | 0.65503 | 1.121106 | 0.775291 | 5.75E-07 | 0.000119 |
| TRAPPC3L | 0.164103 | 0.031343 | -2.38838 | 0.003681 | 0.040743 |
| AC025271.2 | 0.026817 | 0.01402 | -0.9357 | 0.003968 | 0.042751 |
| NCAM2 | 0.294187 | 0.137574 | -1.09653 | 0.002301 | 0.03095 |
| KRT18P20 | 0.056267 | 0.106409 | 0.919263 | 0.00106 | 0.019436 |
| KRT8P11 | 0.210586 | 0.398368 | 0.919693 | 0.001891 | 0.02767 |
| AC026410.2 | 0.400118 | 0.691274 | 0.788833 | 9.01E-05 | 0.003986 |
| KRT18P42 | 0.022848 | 0.046772 | 1.033556 | 1.65E-05 | 0.001274 |
| ZFPM2 | 0.903235 | 0.469789 | -0.94309 | 0.000345 | 0.009733 |
| MYH10 | 12.22044 | 7.50678 | -0.70303 | 0.000553 | 0.01322 |
| ANKRD33B | 1.934797 | 0.929279 | -1.058 | 0.000286 | 0.008538 |
| AL049775.1 | 0.148799 | 0.042769 | -1.79872 | 0.000894 | 0.017519 |
| NDUFA1 | 109.5673 | 187.7159 | 0.776733 | 3.54E-18 | 5.34E-14 |
| PAK3 | 0.290198 | 0.153433 | -0.91943 | 0.001144 | 0.020319 |
| SEM1 | 9.053099 | 15.07876 | 0.736034 | 7.75E-06 | 0.000744 |
| ST8SIA1 | 0.342438 | 0.193544 | -0.82318 | 9.93E-05 | 0.004255 |
| ATP5MFP5 | 0.473908 | 0.919611 | 0.956417 | 1.13E-05 | 0.001011 |
| AARD | 0.266696 | 0.120004 | -1.15212 | 0.001355 | 0.022352 |
| KRT18P24 | 0.018293 | 0.038289 | 1.065666 | 0.000807 | 0.016537 |
| AL022162.1 | 0.912179 | 1.546574 | 0.761687 | 0.001053 | 0.019343 |
| AC055874.1 | 0.027406 | 0.011895 | -1.20411 | 0.000591 | 0.013687 |
| FLNA | 222.1657 | 128.1517 | -0.79378 | 1.75E-05 | 0.001333 |
| AC010531.6 | 0.490311 | 0.879937 | 0.843703 | 0.00024 | 0.007644 |
| MIR5572 | 0.146985 | 0.623499 | 2.084716 | 0.001923 | 0.027956 |
| KRT8P3 | 4.279417 | 8.842967 | 1.047116 | 0.001185 | 0.020792 |
| RNU6-26P | 1.039309 | 0.489036 | -1.08761 | 0.001647 | 0.025251 |
| EVC2 | 1.047906 | 0.590709 | -0.82699 | 0.000638 | 0.014372 |
| PRICKLE1 | 0.875741 | 0.507257 | -0.78779 | 0.000934 | 0.017982 |
| AC108451.2 | 0.157029 | 0.649558 | 2.048427 | 0.00043 | 0.011284 |
| PLXNA4 | 0.45506 | 0.25573 | -0.83144 | 0.00027 | 0.008191 |
| TMEM270 | 0.288414 | 0.475328 | 0.720785 | 0.00188 | 0.027569 |
| LINC01238 | 0.272313 | 0.142432 | -0.93499 | 0.004183 | 0.044264 |
| AC116533.1 | 41.61663 | 70.23287 | 0.754986 | 4.48E-09 | 3.38E-06 |
| MTCO3P13 | 0.075624 | 0.225583 | 1.576739 | 0.000259 | 0.007976 |
| RPL39P39 | 0.148267 | 0.242509 | 0.709842 | 0.003254 | 0.038072 |
| COX5AP2 | 0.083183 | 0.136062 | 0.709895 | 0.00071 | 0.015267 |
| NUP62CL | 1.878641 | 3.193851 | 0.765608 | 3.18E-05 | 0.001999 |
| MT-TP | 1563.711 | 2682.804 | 0.778767 | 0.000209 | 0.007006 |
| AL359555.4 | 0.010594 | 0.006491 | -0.70672 | 0.001694 | 0.025712 |
| CASC8 | 0.670569 | 1.410904 | 1.073162 | 0.00029 | 0.008581 |
| KRT8P45 | 1.506731 | 3.152389 | 1.065023 | 0.001379 | 0.0225 |
| KRT18P49 | 0.021506 | 0.065348 | 1.603396 | 0.000181 | 0.006351 |
| KRT18P67 | 0.02528 | 0.050254 | 0.991237 | 0.000542 | 0.013082 |
| INHBA-AS1 | 0.12447 | 0.074514 | -0.74022 | 0.003015 | 0.036488 |
| TRABD2B | 0.639643 | 0.331868 | -0.94666 | 0.000158 | 0.005756 |
| DOCK8 | 2.738026 | 1.558231 | -0.81323 | 0.005104 | 0.049842 |
| VCAM1 | 7.077583 | 3.028428 | -1.22469 | 0.001989 | 0.028507 |
| KRT19P1 | 0.651425 | 1.315014 | 1.013407 | 0.004947 | 0.048754 |
| RNA5SP435 | 0.281955 | 0.142352 | -0.986 | 0.000135 | 0.005159 |
| NR2F2-AS1 | 0.143252 | 0.084278 | -0.76534 | 0.002189 | 0.030075 |
| KRT18P15 | 0.238725 | 0.39332 | 0.720351 | 0.001185 | 0.020792 |
| KRT8P49 | 0.109049 | 0.20501 | 0.910712 | 0.000833 | 0.016798 |
| DCLK1 | 0.529717 | 0.311081 | -0.76793 | 0.003527 | 0.039689 |
| APCDD1L | 1.879494 | 1.027602 | -0.87106 | 0.002367 | 0.03139 |
| AL009178.3 | 0.108333 | 0.19609 | 0.856048 | 0.002872 | 0.035313 |
| MTRNR2L10 | 0.415149 | 0.690635 | 0.734292 | 1.75E-05 | 0.001333 |
| TSHZ3 | 2.302286 | 1.331658 | -0.78984 | 0.000708 | 0.015222 |
| FLRT3 | 5.39687 | 3.141948 | -0.78046 | 0.00234 | 0.031249 |
| NOX1 | 2.314327 | 3.959536 | 0.774738 | 0.003721 | 0.041061 |
| RUNX2 | 3.198574 | 1.796114 | -0.83255 | 2.36E-06 | 0.0003 |
| GDPD2 | 0.603778 | 1.964417 | 1.70201 | 4.91E-05 | 0.00271 |
| RPL39P33 | 0.101952 | 0.190715 | 0.903529 | 0.001194 | 0.020867 |
| MPDZ | 1.887974 | 1.04317 | -0.85586 | 6.44E-05 | 0.003195 |
| LINC02152 | 0.130042 | 0.015095 | -3.10683 | 0.000759 | 0.015976 |
| AC022098.3 | 0.110949 | 0.207135 | 0.900667 | 0.000768 | 0.016082 |
| NAV3 | 0.55359 | 0.233723 | -1.24401 | 0.00087 | 0.01724 |
| SCML2P2 | 0.204917 | 0.112609 | -0.86372 | 0.000677 | 0.014894 |
| AC124014.1 | 0.201196 | 0.089365 | -1.17081 | 0.002816 | 0.034998 |
| TRAJ6 | 0.328059 | 0.154142 | -1.0897 | 0.00259 | 0.033216 |
| LIFR | 2.41788 | 1.397328 | -0.79107 | 7.31E-05 | 0.003506 |
| MTND1P23 | 66.00711 | 123.5286 | 0.904152 | 0.000642 | 0.014397 |
| THSD7B | 0.655051 | 0.160647 | -2.02771 | 0.000578 | 0.013535 |
| NBEA | 0.895243 | 0.464995 | -0.94506 | 9.01E-05 | 0.003986 |
| SDK2 | 2.914886 | 1.379679 | -1.07911 | 0.00029 | 0.008581 |
| AC133552.4 | 0.371044 | 0.20819 | -0.83369 | 0.000415 | 0.01093 |
| PHBP13 | 0.059505 | 0.03593 | -0.72784 | 0.003858 | 0.042068 |
| AL590235.2 | 0.033932 | 0.079258 | 1.223906 | 0.001584 | 0.024607 |
| ZFHX4 | 0.846541 | 0.476935 | -0.82779 | 0.003863 | 0.042069 |
| BNC2 | 0.847257 | 0.474402 | -0.83669 | 0.002213 | 0.030195 |
| NPTX2 | 2.854059 | 1.176957 | -1.27795 | 0.002871 | 0.035307 |
| SNRPEP6 | 0.02914 | 0.0521 | 0.838276 | 0.003971 | 0.042767 |
| CHST11 | 8.213791 | 3.934195 | -1.06198 | 5.87E-05 | 0.003018 |
| RPS26P47 | 3.139698 | 5.624326 | 0.841055 | 0.003032 | 0.036556 |
| BACH2 | 0.831489 | 0.493757 | -0.7519 | 0.002327 | 0.031117 |
| GDF10 | 0.1431 | 0.055543 | -1.36534 | 0.000967 | 0.018277 |
| SLIT2 | 1.314722 | 0.608502 | -1.11142 | 0.000251 | 0.007829 |
| ABCA9 | 0.408822 | 0.210247 | -0.95939 | 0.003565 | 0.039939 |
| ZNF853 | 2.53678 | 1.492151 | -0.76561 | 0.004317 | 0.045129 |
| KRT18P3 | 0.065826 | 0.137201 | 1.059566 | 0.000189 | 0.006535 |
| AL139317.4 | 0.161215 | 0.086515 | -0.89797 | 0.000603 | 0.013814 |
| AC025181.1 | 0.224274 | 0.378083 | 0.753439 | 0.001185 | 0.020792 |
| SLC25A5P6 | 0.059383 | 0.103917 | 0.807306 | 5.32E-08 | 2.10E-05 |
| MEPE | 0.032196 | 0.017447 | -0.88392 | 0.001358 | 0.022373 |
| KRT18P28 | 0.106885 | 0.19222 | 0.846695 | 0.002392 | 0.031635 |
| ITGA4 | 2.143131 | 1.306602 | -0.7139 | 0.000436 | 0.011397 |
| AL356441.2 | 0.026601 | 0.046532 | 0.806767 | 8.33E-05 | 0.003807 |
| AC008957.3 | 0.251948 | 0.138979 | -0.85826 | 0.001735 | 0.026055 |
| EVC | 4.851829 | 2.592556 | -0.90415 | 1.92E-05 | 0.001413 |
| ZBTB16 | 1.399462 | 0.60174 | -1.21766 | 0.003701 | 0.040917 |
| C1QTNF8 | 0.011516 | 0.037631 | 1.708271 | 0.003417 | 0.038956 |
| THSD4 | 3.521519 | 2.129305 | -0.72582 | 0.000159 | 0.005788 |
| CSPG4 | 17.13925 | 8.831602 | -0.95656 | 0.002226 | 0.030282 |
| DLG3-AS1 | 0.170283 | 0.315753 | 0.89086 | 0.000462 | 0.01184 |
| TIMM8BP2 | 0.300567 | 0.502591 | 0.741698 | 0.000592 | 0.013704 |
| TRBV26OR9-2 | 0.194373 | 0.094296 | -1.04355 | 0.000208 | 0.007006 |
| CACNA2D1 | 3.777641 | 1.849565 | -1.0303 | 0.002327 | 0.031117 |
| VSTM4 | 1.708454 | 0.921867 | -0.89006 | 0.002871 | 0.035307 |
| AC080129.2 | 0.387501 | 0.687847 | 0.827887 | 0.002326 | 0.031117 |
| AC092991.1 | 0.113434 | 0.206001 | 0.8608 | 0.002344 | 0.031271 |
| SMIM22 | 3.435192 | 6.125823 | 0.834513 | 0.002778 | 0.034719 |
| LRRC18 | 0.049467 | 0.020623 | -1.26219 | 0.00444 | 0.045846 |
| AC104843.1 | 0.651706 | 1.106338 | 0.763499 | 1.46E-05 | 0.001167 |
| C4A | 3.06692 | 1.459993 | -1.07083 | 0.001513 | 0.023894 |
| HES6 | 3.920307 | 7.551449 | 0.945787 | 0.000923 | 0.017882 |
| AC026316.1 | 0.158521 | 0.270853 | 0.772837 | 3.60E-05 | 0.002159 |
| AC234778.1 | 0.240861 | 0.418725 | 0.797801 | 2.32E-06 | 0.000298 |
| KISS1 | 0.328509 | 0.845262 | 1.363464 | 0.000939 | 0.018055 |
| AC012511.1 | 0.148361 | 0.07542 | -0.9761 | 0.002815 | 0.034998 |
| PGR | 0.202879 | 0.109144 | -0.89438 | 0.00024 | 0.007644 |
| EMX1 | 0.363898 | 0.746159 | 1.035951 | 0.002717 | 0.03422 |
| AC006157.1 | 0.128863 | 0.275663 | 1.097068 | 0.001892 | 0.02767 |
| NHSL2 | 0.842877 | 0.493391 | -0.77259 | 0.000464 | 0.01184 |
| FIGN | 0.945088 | 0.274 | -1.78627 | 5.95E-05 | 0.003035 |
| LINC01063 | 0.530571 | 0.980517 | 0.885997 | 0.003166 | 0.037417 |
| RPL36AP51 | 0.098891 | 0.181414 | 0.875375 | 7.60E-05 | 0.003588 |
| SRPX | 6.835755 | 3.639333 | -0.90943 | 7.00E-05 | 0.003399 |
| AC010531.2 | 0.078917 | 0.154764 | 0.971659 | 0.000213 | 0.007098 |
| AC002550.1 | 0.03384 | 0.063327 | 0.90409 | 8.59E-05 | 0.003879 |
| AC007370.1 | 0.039247 | 0.076812 | 0.968759 | 0.002468 | 0.032266 |
| H2BC14 | 0.574397 | 0.951509 | 0.728169 | 0.001123 | 0.02014 |
| CKMT1B | 4.038061 | 6.641975 | 0.71795 | 8.33E-07 | 0.000145 |
| AFF3 | 0.317133 | 0.153372 | -1.04806 | 0.001271 | 0.021578 |
| TLDC2 | 0.781827 | 1.348374 | 0.7863 | 5.91E-05 | 0.003024 |
| AC023449.1 | 0.169104 | 0.301733 | 0.835366 | 0.001724 | 0.025984 |
| ABCA6 | 0.390023 | 0.205691 | -0.92308 | 0.001766 | 0.026359 |
| MIR200CHG | 5.006308 | 9.477053 | 0.920691 | 0.003967 | 0.042751 |
| AL162376.1 | 0.207346 | 1.331185 | 2.682601 | 0.000677 | 0.014893 |
| MCF2L2 | 0.197882 | 0.118114 | -0.74447 | 0.00455 | 0.046407 |
| CKMT1A | 4.006705 | 6.870267 | 0.77795 | 7.19E-08 | 2.58E-05 |
| AC005670.1 | 0.09439 | 0.171447 | 0.861054 | 0.002222 | 0.030282 |
